# Sub-daily virus sampling at the Bermuda Atlantic Time Series reveals diel and depth-structured population dynamics without community-level shifts

**DOI:** 10.1371/journal.pbio.3003474

**Published:** 2026-03-06

**Authors:** Alfonso Carrillo, Emily Hageman, Lauren Chittick, Anna I. Mackey, Kimberley S. Ndlovu, Funing Tian, Naomi E. Gilbert, Daniel Muratore, Dean Vik, Gary R. LeCleir, Christine Sun, Ho B. Jang, Ricardo R. Pavan, Joshua S. Weitz, Steven W. Wilhelm, Matthew B. Sullivan

**Affiliations:** 1 Department of Microbiology, The Ohio State University, Columbus, Ohio, United States of America; 2 Centre of Microbiome Science, The Ohio State University, Columbus, Ohio, United States of America; 3 College of Veterinary Medicine, Midwestern University, Glendale, Arizona, United States of America; 4 EMERGE Biology Integration Institute, The Ohio State University, Columbus, Ohio, United States of America; 5 Lawrence Livermore National Laboratory, Livermore, California, United States of America; 6 Department of Microbiology, The University of Tennessee, Knoxville, Tennessee, United States of America; 7 School of Biology, Georgia Institute of Technology, Atlanta, Georgia, United States of America; 8 Santa Fe Institute, Santa Fe, New Mexico, United States of America; 9 Korea Virus Research Institute, Daejeon, South Korea; 10 Institute of Health Computing, The University of Maryland, College Park, Maryland, United States of America; 11 Strategic Partnership: MPowering the State, The University of Maryland, College Park, Maryland, United States of America; 12 The Infectious Disease Institute, The Ohio State University, Columbus, Ohio, United States of America; Monash University, AUSTRALIA

## Abstract

Ocean microbes contribute to biogeochemical cycles and ecosystem function, but they do so under top-down pressure imposed by viruses. While viruses are increasingly understood spatially and beginning to be incorporated into predictive modeling, high-frequency ocean virus dynamics remain understudied due to methodological challenges. Here we sampled stratified Bermuda Atlantic Time Series (BATS) waters for 112 hours at sub-daily 4- (surface) or 12- (deep chlorophyll maximum) hour intervals, purified viral particles from these samples, sequenced their metagenomes, and used the resulting data to characterize high-frequency virus community dynamics. Aggregated community diversity metrics changed with depth, but were not statistically significant temporally at a fixed location. However, finer-scale population-level analyses revealed both depth and temporal change, including physicochemical depth-driven differences and, in surface waters, thousands of viral populations that exhibited statistically significant diel rhythms. Statistical analyses revealed three main archetypes of temporal dynamics that themselves differed in abundance patterns, host predictions, viral taxonomy, and gene functions. Among these, highlights include viruses resembling an archetype with a night peaking pattern in activity that include an over-representation of viruses that putatively infect *Prochlorococcus*, a phototrophic cyanobacteria. Together, these efforts provide baseline community- and population-scale short-time-frame observations relevant to future climate state modeling.

## Abstract 2

Viruses are often studied in disease settings, but in the ocean they outnumber cells and shape ecosystems, yet their dynamics remain poorly understood. In this study, high-resolution sampling of blue-ocean waters revealed little community-level change but extensive depth- and diel-driven population dynamics beneath the surface.

## Introduction

The oceans play key roles in global biogeochemical cycles, including buffering against human-accelerated climate change [1]. However, this buffering is self-limiting—ocean biogeochemistry is directly impacted by climate change as warming surface waters increase seasonal stratification [2] and see reduced nutrient concentrations. These impacts are particularly important over the vast oligotrophic ocean regions that cover approximately 30%–50% of the Earth’s ocean surface [3,4]. Such altered surface ocean functions are hypothesized to impact the ocean’s ability to absorb atmospheric carbon dioxide and sink surface-produced carbon to the deep sea via the biological carbon pump. While one-third of anthropogenic carbon dioxide released into the atmosphere is absorbed into surface waters via mass action [1], its fate is dictated by plankton—including bacteria, archaea, and microbial eukaryotes that serve as the base of the food web and drive the biological carbon pump.

Viral roles in these ocean biological carbon pump processes are recently being revisited. For decades, the viruses that infect these cells were thought to keep carbon in the dissolved phase by lysis resulting in remineralization—a process known as the viral shunt [5]. However, viral lysis may also generate sticky aggregates that sink out of the photic zone. This “viral shuttle” has been hypothesized as a mechanism that reduces the retentiveness of the microbial loop [6–10]. Recent work leveraging machine learning, statistical modeling, and global ocean datasets from the *Tara* Oceans expeditions provides evidence in support of this hypothesis given that globally ocean carbon flux is best predicted by viral abundances—even more so than abundances of bacteria, archaea, or eukaryotes [11]. In parallel, viral infection of microbial hosts has shown viruses to reprogram infected cells (termed “virocells,” [12]) into entities that are metabolically and biogeochemically different from their uninfected sister cells [13–16]. This reprogramming can also apply when cells mutate to successfully defend themselves against virus attack. Towards this, recent experimental work shows spontaneous virus-resistant marine bacterial mutants alter their carbon substrate utilization, metabolite secretion, and aggregation and sinking rates in ways that will drastically alter these cells’ ecosystem inputs and outputs [17]. Thus, viruses can alter a cell’s ecosystem outputs well beyond simple lysis.

Early work on Kill-the-Winner models showed how viruses infecting a single microbial host could enable fluctuating host diversity through negative frequency-dependent selection [18–20]. These models have been extended to include increased complexity - spanning interaction networks, community dynamics, and feedbacks with ecosystem functioning [21–29]. However, a major obstacle to extending these models to the Earth System scale is the lack of observational data to guide model incorporation, with sampling challenges resulting in relatively little being known about marine microbial and viral temporal dynamics. Some time series sampling efforts have assessed, at monthly resolution, abundance changes in bacteria and archaea (using PCR-amplicons and/or metagenomic sequencing), and picoeukaryotes (via flow cytometry)—and this has been done for years (e.g., 7 year Bay of Banyuls [30]) or even decades (e.g., >30 years Hawaii Ocean Time (HOT) Series [31]). However, these studies largely excluded viruses, except as bycatch in early low-resolution metagenomic surveys [32,33]. Other time series similarly surveyed bacteria, archaea, and picoeukaryotes over long time scales monthly, while also including viruses (e.g., >15 years at San Pedro Ocean Time (SPOT) Series [34–36]). Although they are based on amplicon-based approaches [37], these studies nonetheless provide strong evidence of seasonality whereby bacteria, archaea, and picoeukaryote abundances cycle yearly and then return to (nearly) the same state with only slight year-to-year baseline shifts. This is additionally supported by more recent, monthly viral metagenomics analyses such as those from the SPOT Series that show seasonal dynamics in viral communities along with multi-year stability across five years. Additionally, this metagenomic lens revealed continual within-population genetic turnover consistent with Red Queen-like strain dynamics [38] thought to emerge from Kill-the-Winner ecological interactions [18–20].

At a much finer temporal resolution, some studies have explored diurnal rhythms in ocean systems for microbes and their viruses. Sub-daily timescale metatranscriptome-derived microbial and viral transcript measurements have helped identify strong diel rhythms of gene expression along with temporal relationships between gene expression and metabolite concentration [39–43]. Observations of transcript activity between viruses and their hosts have shown that viruses can often coordinate their activity with host diel cycles, responding to environmental cues in synchrony with their hosts [41–42]. Other work has taken more targeted approaches by focusing on one group, cyanobacterial viruses (or cyanophages), and revealed transcriptional rhythms and adsorption rates in the field that laboratory studies then linked to diurnal photosynthetic activity [44]. Along with this, amplicon-based marker gene fragment patterns (e.g., terminal restriction fragment length polymorphism) have similarly been used to target T4-like viruses to reveal daily dynamics over the course of 38 consecutive days that correlate virus operational taxonomic units (vOTUs) with bacterial OTUs to document short-term variation in both microbial and viral communities [35]. Together, these studies demonstrate clear sub-daily dynamics, but are limited in that they again either catch viruses as a byproduct of microbial sampling (e.g., prokaryotic metatranscriptomics) or target specific virus groups (e.g., cyanophages or T4-like viruses) rather than community-wide signals often doing so with highly degenerate primer strategies that might confound quantitative data generation.

In the decade since these studies, viral metagenomics [37,45] and our understanding of virus population biology [46–48] have matured to the point that there is opportunity to study viruses at increasingly high taxonomic and temporal resolution, even over a short timescale. The Bermuda Atlantic Time Series (BATS)—a sentinel model ecosystem for “future oceans” due to its increased stratification and acidification [49]—has been surveyed via DNA staining and epifluorescence microscopy to quantify virus and microbe abundances and found seasonality over a decade of sampling from 2000 to 2009 [50]. While BATS samples were targeted by these extensive SYBR-stained virus abundance data and early sequencing technology was applied to a single virus sample [51], no genome-resolved virus sequencing data were available at BATS. This prevents ecological inferences like the kinds of viruses, their hosts, their functions, and their metabolic reprogramming capacities. Towards this, recent work employed time-resolved metagenomics at BATS and then compared viral population-based abundances inferred from those captured in paired cellular and viral fraction metagenomes. This revealed that viral fraction population data represents the integral of cellular fraction infections and multiple days of viral turnover [52], but left open questions about viral community and population-level changes through time.

Here, we build upon these prior efforts by establishing a 112-hour time series—sampled every 4 hours in surface waters and, every 12 hours at the deep chlorophyll maximum (DCM)—of virus metagenomic data at BATS during late seasonal stratification as a proxy for future climate-change-impacted oceans. This resulted in detailed, sub-daily insight into community- and population-level viral dynamics for 48,428 viral populations at BATS.

## Results

### Assembly of a high quality and detailed viral reference database at BATS

**Fig 1:**
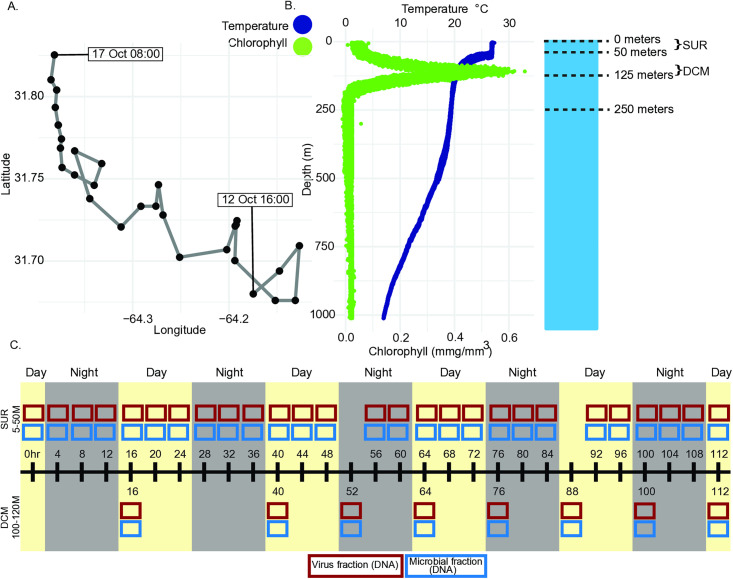
Field site location, depth profiles, and sampling schematic. **A.** The Bermuda Atlantic Time Series is located in the Sargasso Sea portion of the Atlantic Ocean. Lagrangian sampling track for data collection is shown with each black point representing a CTD-cast starting on Oct. 12th, 2019 at 16:00 local time (GMT-3) and ending on Oct. 17th, 2019, at 08:00. The data underlying this Figure can be found in S1 Table. **B.** Depth profile of chlorophyll (green) and temperature (blue) down to 1,000 meters depth, and ship schematic to emphasize key depths in the water column including ship (0 m), surface (0–50 m), and DCM (105–120 m). The data underlying this Figure can be found in S1 Data. **C.** Sampling schematic to show the depth (surface or SUR vs. deep chlorophyll maximum or DCM), frequency (every 4 and 12 hours for surface and DCM, respectively), size fraction (virus red boxes or cellular blue boxes), and time of day (yellow or gray background shading) of each sample taken.

To estimate sub-daily virus population-level dynamics, we established a high-resolution dataset from surface (SUR) and deep-chlorophyll maximum (DCM) waters sampled at BATS between October 12−17, 2019 (Fig 1A–1C). A total of 39 virus concentrates were prepared via chemical flocculation [53] (see Methods) of samples collected every 4 (SUR waters) or 12 (DCM waters) hours over a 112-hour time course (Fig 1C). To follow the same parcel of water through the time-course, sampling was done in a Lagrangian manner using a surface buoy with an underwater drogue (at 30 m depth) to ‘track’ the water for the length of the study. Sampling started at 16:00 local time (GMT-3) and depths were determined with each collection from water column profiles of temperature and salinity collected by the ship’s CTD system as described previously [54].

The resultant virus-fraction concentrates were resuspended in a buffer, DNA extracted, sequencing libraries that targeted double-stranded DNA viruses only were prepared, and then short-read sequenced (see Methods) to an average depth of 147M reads per sample. The resultant 39 virus metagenomes were assembled (see Methods) into 1.48B contigs (≥10 kb) and 228,013 of these were conservatively identified as viral (using the VirSorter 2 Standard Operating Procedure VS2_SOP [55,56]). These virus contigs were then clustered into 48,428 viral populations using community consensus cut-offs of 95% average nucleotide identity over 80% of the contig coverage [47,48,57] with an average genomic length captured of 10.28 kbp. These viral populations were also compared with GeNomad [58] which revealed that 94.92% of the viral populations were shared between both methods. With 99.29% of the VirSorter2 viral populations being also found in GeNomad. Given these results, we find that GeNomad is identifying the same viruses as VirSorter2 as well as additional ones, as there are no clear guidelines on how to efficiently add the results from different viral identification tools [59] we will use VirSorter2’s output alone for all downstream analyses. While other ocean datasets reflect more dispersed sampling schemes, such as the deeply sequenced GOV2 dataset that spread its sequencing across 79 sites [47], here we provide a more focused sampling of the BATS site to maximally identify virus populations at this site. Indeed, this provides us with many more captured viruses at a single site (compare 48,428 with an average of 2,083 viral populations per site in GOV2), at a similar genomic coverage per population (compared 10.28 kb versus GOV2’s average genomic length of 10.17 kbp [47]). Taxonomically, these populations are thought to represent approximately species-level taxa as prior studies inferred from (i) gene flow, selection, and genomic fixation indices [46], (ii) host range-based phenotypic variation in heterotrophic viruses [60], and (iii) natural breaks in sequence similarity for nearly 500K virus populations in GOV2 [47].

### Community level diversity significantly varies with depth, but not through 112-hour time-course

**Fig 2:**
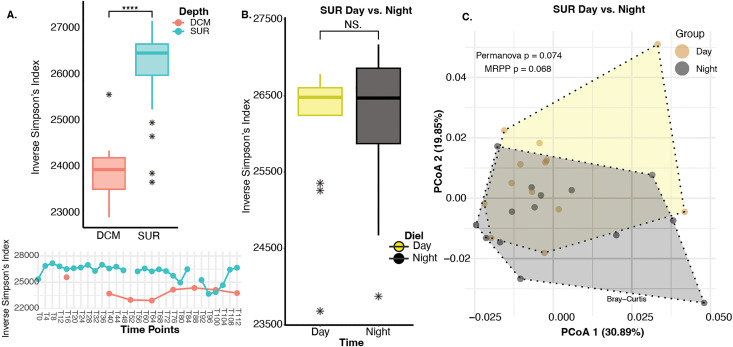
Community-level statistics across water depths and times of sampling. **A.** Inverse Simpson’s Index box plot showing that virus community diversities were statistically significantly different (Wilcoxon Rank Sum *P* = 0.000028) between SUR (blue points) vs. DCM (pink points). Line graph below depicting alpha diversity (Inverse Simpson’s) changes across time points for SUR (blue points) and DCM (pink points) samples. The data underlying this Figure can be found on our zenodo repository (see Data availability) within file all-meancov.txt. **B.** Inverse Simpson’s Index box plot of day (yellow points) vs. night (black points) for SUR samples showing that virus community diversities were not significantly different (Wilcoxon Rank Sum *P* = 0.62, denoted as NS in the figure). The data underlying this Figure is the same as in Fig 2A. **C.** Principal coordinate analysis of Bray-Curtis dissimilarity distances shows that day vs. night virus community dissimilarities were not significantly different (Multi-Response Permutation Procedure [MRPP], *p* = 0.068). Shaded polygons outline day and night groups. The data underlying this Figure is the same as in Fig 2A.

We first sought to assess how community-level diversity changed with depth and across our high-frequency time course (see Methods). For depth-resolved comparisons, we hypothesized that viral diversity would differ significantly between surface (SUR—sampled from 5 m) and DCM samples, due to the inherent differences in physicochemical features such as temperature, and light penetration that also impact biology (e.g., chlorophyll as a proxy for phototrophic biomass) ([61–63]; Figs 1B, S2, and S3). Indeed, we found that virus community alpha diversity (Inverse Simpson’s Index) was significantly higher in SUR versus DCM waters (Wilcoxon Rank Sum *P* = 0.000028) (Fig 2A), and this remained stable for nearly all time points throughout the 112-hour time course (Fig 2A). Along with this, we found viral community beta diversity (Bray-Curtis) to be significantly different between SUR and DCM samples (S3 Fig). These findings complement other daily observations at BATS where extracellular viral abundances had clear depth-specific differences inside and outside the surface mixed layer [52]. Additionally, we evaluated Inverse Simpson’s diversity changes within SUR versus DCM depths of water columns sampled throughout the global oceans alongside our own. This revealed that BATS had higher alpha diversity in SUR as opposed to the DCM, contrary to other previously sampled regions ([47], S4 Fig).

We also assessed the impact of the diel cycle on diversity proxies in the surface samples only. Specifically, we evaluated how alpha- and beta-diversity metrics varied between nighttime (20:00) and daytime (08:00). Although some microorganisms possess unique circadian rhythms and dependencies on the light-dark cycle [64], community-wide virus diversity metrics were not significantly different whether inferred at the level of alpha diversity (Inverse Simpson’s diversity metric; Wilcoxon Rank Sum *P* = 0.62; Fig 2B) or beta diversity (PCoA ordination of Bray-Curtis dissimilarity measures; MRPP *P* = 0.068; Fig 2C). Thus even though day/night light intensity and microbial taxa change during this 112-hour sampling [54], our findings show that aggregate viral community diversity measures do not change. Our 2019 BATS viral sampling expands upon prior 2017 BATS viromic sampling [52] by increasing resolution (4- versus 12-hour sub-daily time scale) and adding 20× fold more viruses at a single site (48K versus 2.3K). We reasoned then that these significant dataset improvements provided a better opportunity to assess high-resolution virus community dynamics at BATS.

### Viral populations and potential metabolic gene repertoire vary across depth

Given that the viral depth-related differences shown above were explored at the community-level, we continued by exploring depth-related changes at the virus population level. First, we predicted hosts for our 48,428 virus populations to assess their patterns across the two depths sampled. Notably, we used aggregate *in silico* prediction strategies (see Methods) and maximized our ability to predict hosts (prior benchmarking suggests host prediction improvement of 25% for most systems [65] by augmenting the standard database available with 89 high-quality metagenome-assembled genomes (MAGs) from prior BATS work [52]. This resulted in predicted hosts for 11,814 (24.39%) of our 48,428 total populations, a large improvement on the 6 (0.26%) of 2,301 total viral populations whose hosts could be predicted in that prior study [52].

**Fig 3:**
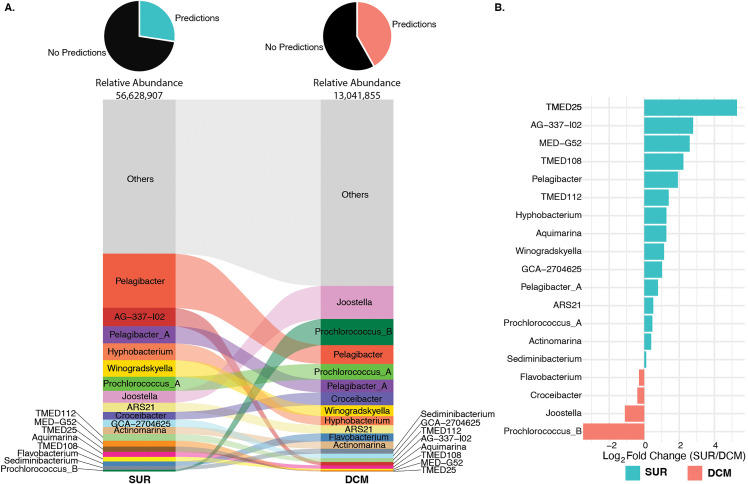
Viral population hosts across depths. **A.** Alluvial plot of predicted hosts (genus-level ranks) for viral populations present at each depth. Height of row is based on abundance of genera and genera that make up less than or equal to 1% of the total abundances are grouped as “Others”. Alluvial is ordered from most abundant at top to least abundant at bottom. The pie chart on top of each column indicates the percent of viral populations with host predictions in each group with the total relative abundance of the category (SUR/DCM) below. The data underlying this Figure can be found in our zenodo repository (see Data availability) within file all-meancov.txt and combined_host_prediction_to_genome_m90.csv. **B.** Bar graph showing the Log_2_ fold change of host predictions across depths. Log_2_ fold change is based on abundances of genera that make up greater than 1% of the total abundances in the dataset. The data underlying this Figure is the same as that of Fig 3A.

SUR versus DCM depth comparisons revealed some populations were shared across depths, whereas others exclusive. As expected, hosts whose virus populations were better sampled displayed abundance changes across the depths. For example, viruses that infect heterotrophic bacteria such as *Pelagibacter* (SAR11 clade I), *Pelagibacter_A* (SAR11 clade II), and *AG-337-I02* (SAR11 clade V) were among the most represented host predictions in SUR, with *AG-337-I02* and *Pelagibacter* ~4-fold or higher in abundance at the surface compared to DCM (Fig 3A and 3B). This SUR enrichment signal of SAR11 clade infecting viruses parallels SAR11 bacteria being one of the most abundant heterotrophic surface bacteria found in the Sargasso Sea, dominated by SAR11 clades I and II in BATS SUR waters [66,67]. As well, a higher proportion of viruses found in DCM samples were predicted to infect phototrophic *Prochlorococcus*, with *Prochlorococcus_B* the dominant *Prochlorococcus* ecotype and having greater than 6-fold abundance in DCM compared to SUR (Fig 3A). This signal is consistent with *Prochlorococcus_B* being a low-light ecotype that prefers lower depths, like the DCM, at BATS [50,68].

Looking further, we wondered whether viruses across these depths contained any metabolic genes of interest (e.g., auxiliary metabolic genes, or AMGs), and how these would fit into measured microbial metabolisms within the Sargasso Sea. [12–15,69]. We sought to uncover any AMGs in viruses unique to either depth environment. To ensure that genes were not misattributed as AMGs, we adopted a highly conservative approach for curating our AMG catalog, as used previously (see Methods; [70], which resulted in only ~0.05% (349) of 684K annotated genes passing our conservative filtering. Beyond this conservative filtering, we did not conduct any manual curation or further validation.

**Fig 4:**
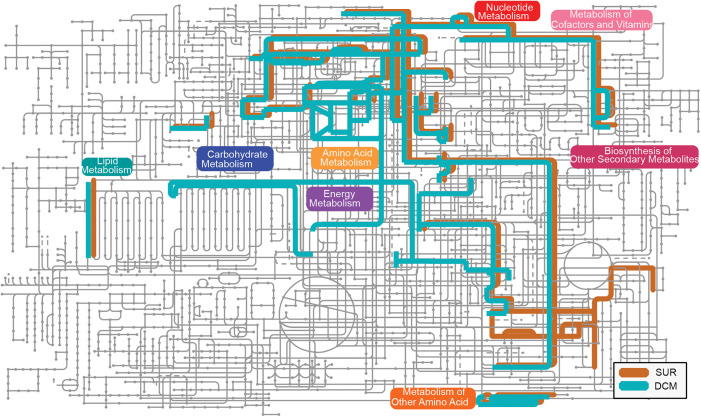
Metabolic pathways found in viruses across depth gradients. KEGG map was made on iPath (v3) under map selection “Metabolic Pathways” using data from S5 Table and shows pathways for AMGs present in viruses across depth gradients. Color of line is based on what depth the virus containing the AMG was found in, with gray lines indicating lack of AMGs relevant in that pathway for either group. This KEGG map is a qualitative analysis depicting which pathways identified AMGs belong to, with lines indicating pathways with relevant AMGs present. Thickness of the line is the same throughout figure to highlight presence of pathways.

Many of the putative AMGs belonged to pathways enriched in one depth over the other (Fig 4). Among these, cobalamin biosynthesis was SUR-enriched and of particular interest given that *Prochlorococcus* and *Synechococcus* require cobalt for microbial growth [71,72] and comparative *Prochlorococcus* genomics has documented their genetic machinery for the synthesis and use of cobalt-bearing cofactors (cobalamins) [71]. Considering this, we hypothesized it was possible that abundant phototrophic viruses in the viral community harbor AMGs that assist in cobalamin biosynthesis. Assessing our data, however, we found a single gene in the cobalamin biosynthesis pathway (K09882, *cobS*) in a viral population exclusively within SUR (S5 Table). Host prediction suggested that this population infects *SAR86A*. The *SAR86A* prediction is curious given that this lineage is not known to possess any genes involved in cobalamin biosynthesis.

Taking instead, a host-focused perspective on AMG depth-related patterns, we observed the following. First, we explored virus populations predicted to infect *Pelagibacter* (formerly known as SAR11), i.e., pelagiphages [73], which revealed 3 AMGs among them including *phoH*, K06217 (1 viral population) and 2OG-Fe(II) oxygenase, K07336 (2 viral populations). Of these, *phoH* is known to be present in both cyanophages [74] along with heterotrophic phages including pelagiphage [75]. Phylogenetic analysis of the *phoH* gene in phages has been shown to vary greatly based on the phage host while the role of *phoH* remains unclear as expression of the gene has been shown to vary within different phage-host pairs [76]. The 2OG-Fe(II) oxygenase AMG, however, is a much more well-known gene in pelagiphages [73]. Curiously, however, these pelagiphage AMGs were depth-specific with the viral populations possessing said genes only being present exclusively in one depth or the other. This can be seen with one of the viral populations predicted to target *Pelagibacter_A* that possessed a 2OG-Fe(II) oxygenase being found only in DCM, while all other pelagiphages with AMGs were found exclusively within SUR. Second, we explored AMG depth-related signals from *Prochlorococus*, which revealed a photosystem II gene (K02703, *psbA*) found in 7 viral populations exclusive to the DCM, where two populations were predicted to infect *Prochlorococcus_B* and the remaining five were predicted to infect *TMED108, TMED70, MGIIa-K1*, and 2 for unknown hosts. Within these host predictions both *TMED108* and *TMED70* are heterotrophic hosts while *MGIIa-K1* is a member of marine group II *Euryarchaea* [40]. *TMED108* is a member of the Marinisomatota phylum (formerly recognized as Marinimicrobia, Marine Group A, and SAR406) which has traditionally been characterized as heterotrophic microorganisms, although recently has been seen to possess members that demonstrate the capacity to harness light-dependent processes associated with Crassulacean acid metabolism [77] *TMED108* and *MGIIa-K1* are members of widely distributed and ecologically important clades of marine microbes [40,77–79], however, none of these genera have previously been shown to hold the photosystem II gene, psbA. Although both depths had virus populations with *Prochlorococcus_B* predicted as the host, this genus was a common (second most) predicted host in the DCM, 6× more abundant in DCM compared to SUR—all of which underscored its known low-light ecotype adaptations that would do well in DCM.

Finally, though a weaker signal, we highlight the finding that a SUR virus population contained a gene putatively involved in Coenzyme M biosynthesis (K05979, *comB,* 1 viral population), presumably relevant to methanogenic archaea. The coenzyme M biosynthesis signal might represent an understudied link to methanotrophic archaeal carbon metabolism, specifically anaerobic oxidation of methane to carbon dioxide [80,81]. Such methane cycling has previously been shown in oligotrophic North Atlantic Ocean gyres to be important with elevated methane from archaea as well as some bacteria that produce it as a byproduct [82]. Although the virus possessing this AMG had no host predicted, it is known that archaeal viruses outside extremophile conditions are incredibly challenging to predict [83]. We posit that the presence of a coenzyme M biosynthesis gene in a virus contig and its potential relevance to methane and carbon cycling at BATS, invites future targeted work once pelagic ocean archaeal viruses are more readily identifiable and/or increased genomic context becomes available for this gene.

### Diel periodicity analyses reveal population-level viral population dynamics

Though virus community-level alpha and beta-diversity metrics revealed no significant sub-daily temporal dynamics, prior ocean viral community monthly sampling had revealed population-level variation underpinning such community-level stability [38]. Thus, we wondered whether specific populations were in fact dynamic, even at this sub-daily time frame, and if so, whether they had consistent diel cycles that might be detectable given the extensive sequencing available across this high-resolution time-course. To test this, we applied prior methods [84] to statistically detect diel patterns (via RAIN [85] and significance testing; see Methods) among our virus populations. This revealed 3,097 viral populations that were significantly diel rhythmic in SUR (10.68% of SUR viral populations). The lack of detected diel cycling viruses in DCM may be due to reduced sampling which may have limited statistical power to detect diel rhythmicity.

**Fig 5:**
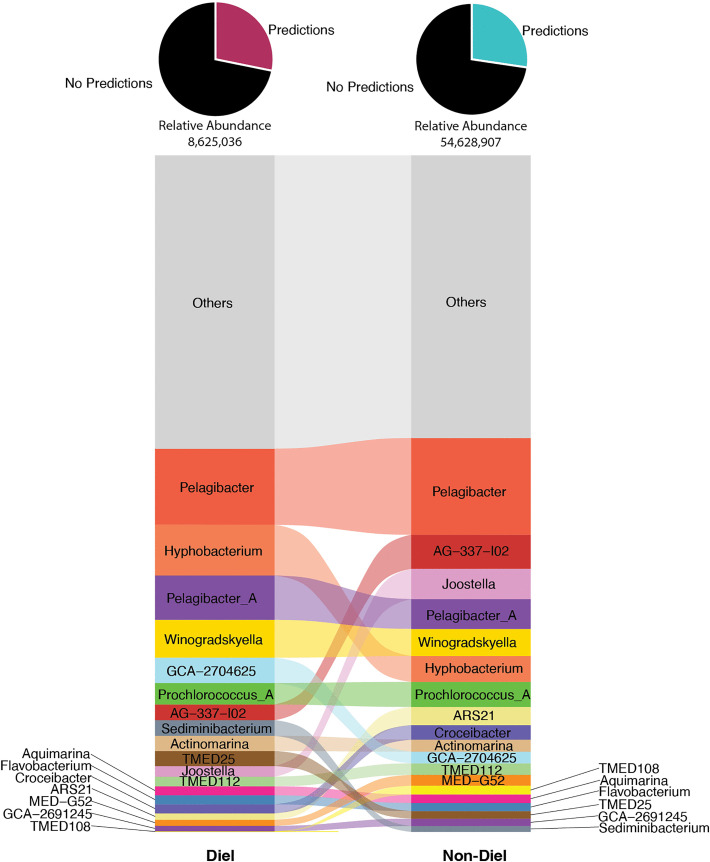
Predicted hosts for SUR viral populations with diel or non-diel rhythmic periodicities. Alluvial plot of predicted hosts (genus-level ranks) for diel and non-diel viral populations present. Height, order, and pie chart are as described in Fig 3. The data underlying this figure can be found in our zenodo repository (see Data availability) in file all-meancov.txt and combined_host_prediction_to_genome_m90.csv.

Focusing on SUR viral populations, we then compared diel versus non-diel SUR viral populations to look for differences at levels of host predictions, viral taxonomy, and gene function. For hosts, the abundances of viral populations for a given predicted host were summed and then assessed across the time course for diel versus non-diel viral populations via RAIN and significance testing as described above (Fig 5). This revealed diel versus non-diel viral abundances for many predicted hosts with *Pelagibacter* being the most abundant targeted taxa across the two categories. However, slight differences were observed for the proportion of viruses targeting taxa like *Hyphobacterium* in the diel group or those targeting *Joostella* in the non-diel group. Such observations are difficult to interpret given that not much is known for either genus regarding their diel behavior in oceans. Similarly when looking at family-level viral taxonomy and their abundances, we observed very little change between diel and non-diel viruses, with slight differences in the enriched proportion of viruses belonging to Caudoviricetes (new family 33 and order 140) for diel and Caudoviricetes (new family 21 and order 134) in non-diel (S5 Fig).

Seeing largely similar host predictions and viral taxonomy for the diel and non-diel viral populations, we assessed their genes for functional gene content signatures. We hypothesized that viruses possessing diel patterns of abundance may possess AMGs that would enable these respective niches. Evaluating our conservatively filtered AMG catalog, we found 18 putative AMGs (belonging to 7 pathways) in diel viruses and 331 putative AMGs (belonging to 39 pathways) in non-diel viruses (S8 and S9 Tables). Most of the genes identified by our conservative filtering methods for diel viruses were shared with non-diel viruses, while most of the non-diel virus genes were not. Among the AMGs shared between diel and non-diel populations, many are likely involved in virus infection to produce genomes and/or particles (e.g., nucleotide metabolism, amino acid metabolism). The only one that was exclusive to the diel viral populations was Coenzyme M biosynthesis (K05979, *comB,* 1 viral population), whereas the non-diel exclusive AMGs included 131 genes involved in 33 pathways (S6–S9 Tables). To summarize their biology, we interpret the non-diel-unique AMGs to represent pathways that contribute to constitutive rather than diel-cycling metabolisms. These constitutive metabolisms include genes involved in carbohydrate and redox metabolism (glucoronate, galactose, glyoxylate, ascorbate), nucleotide metabolism (purine, pyrimidine, degradation), amino acid and membrane metabolism (fatty acids, phospholipids), amino acid and polyamine metabolism (methionine, arginine/polyamines, creatine), cofactor and vitamin biosynthesis (THF, BH₄, B₁₂, biotin), sulfur cycling (sulfate assimilation/reduction) and secondary metabolites (aurachin). While less clear examples are those involved in cell wall/structural sugar biosynthesis (rhamnose, KDO) as dTDP-L-rhamnose biosynthesis which is involved in biosynthesis of terpenoids and polyketides is a pathway not typically found in viruses [86] (S6–S9 Tables).

### Unsupervised learning identifies day- versus night-peaking patterns in diel signals

**Fig 6:**
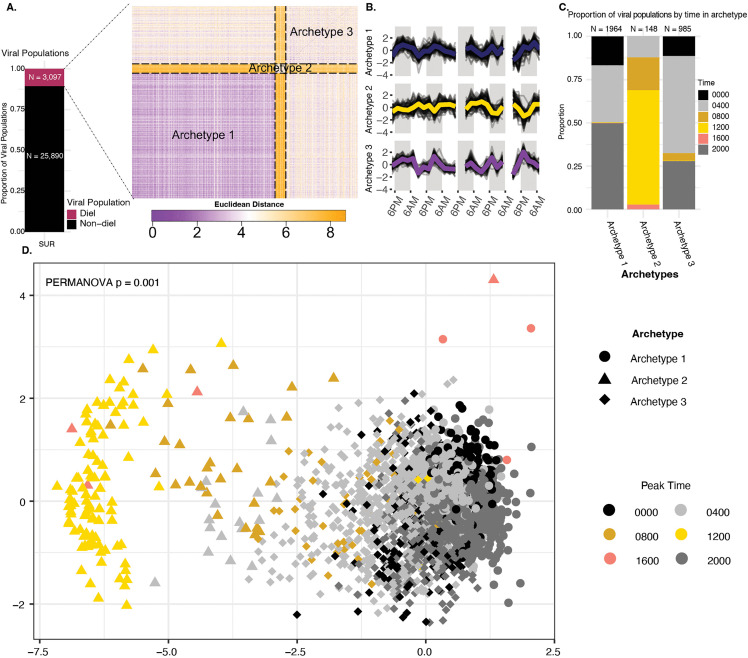
Categorizing diel patterns into three potential archetypes using unsupervised clustering. **A.** Organized pairwise distance matrix for all diel viral populations after clustering based on unsupervised, self-organizing maps (SOMs). Pixels in figure represent the Euclidean distance between the time series of two diel viral populations with purple indicating a small distance (similar time series) and orange indicating a higher distance (less similar time series). Dotted lines form boxes to separate the different cluster boundaries. The data underlying this Figure can be found on our zenodo repository (see Data availability) within file all-meancov.txt **B.** Line graph for time series of viral populations within each cluster. Archetype time series are a combination of all time series in their cluster as defined via the SOM algorithm (midnightblue = Archetype 1; gold = Archetype 2; purple = Archetype 3). A random sample of 145 time series pertaining to each cluster are plotted as dark lines. Data underlying this figure is the same as in Fig 6A. **C.** Proportion for number of viral populations by time in archetype. Columns represent archetype while color represents mean peak time for viral populations across the time series. Data underlying this Figure is the same as in Fig 6A. **D.** NMDS of all diel viral populations colored by calculated peak rank measurement time and given shapes by archetype. Data underlying this Figure is the same as in Fig 6A.

We set out to evaluate the structure of diel rhythmic viral populations given observed variation in amplitude, shape of oscillation, and peak timing. To formally evaluate this, we built on recent efforts to identify viral archetypes in our dataset that may possess recurring temporal patterns, assign groups of viral populations to these archetypes, and assess within- and between-group differences in ecological relevance [84]. With this analytic pipeline [84], we utilized an unsupervised, self-organizing map (SOM) approach to determine whether smaller groups or ‘archetypes’ exist by evaluating the euclidean distances of their abundance across the time series (see Methods). The resultant SOM clustering approach for our 3,097 diel viral populations revealed 3 archetypes that we titled “archetype 1,” “archetype 2,” and “archetype 3” (Fig 6A). Adding to this archetype construction, we calculated the average peak time for individual viral populations as well (see Methods), revealing that most (94.09%) SUR diel viral populations peaked in abundance during the night with the most abundant time point being 04:00 hours, with much less common day peaking viral populations peaking at 12:00 hours.

To assess the archetypes for biological differences, we first evaluated their temporal patterns to connect those possible patterns to host predictions. These temporal patterning analyses revealed that archetypes 1 and 3 possessed night-peaking viral populations and dominated the dataset (95.22% of diel viral populations), whereas archetype 2 possessed primarily day-peaking viral populations and represented only a few percent of the total populations (Fig 6B and 6C). Ordination (NMDS with euclidean distances) revealed a clear separation between viral populations that coincided with time and archetype grouping such that one sees a gradual shift from virus population archetypes from peaking at 00:00 hours to peaking at 12:00 hours (Figs 6D and S6). Because these archetypes required no *a priori* assumptions of sinusoidal patterns nor of preferred phase, these observations represent naturally forming groups that presumably more accurately capture diel rhythmicity across the time course (see also [84]).

**Fig 7:**
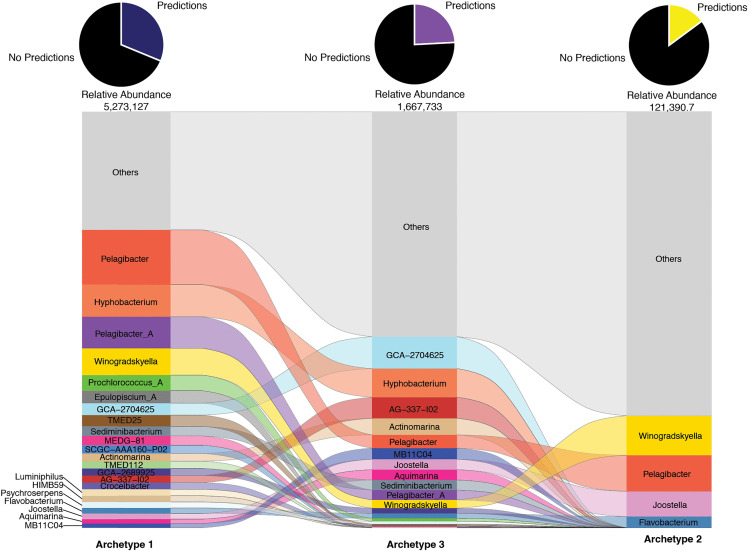
Comparison of diel archetypes host predictions. Alluvial plot of predicted hosts (genus-level ranks) for viral populations present within archetypes. Height, order, and pie chart are as described in Fig 3. The data underlying this figure can be found in our zenodo repository (see Data availability) in file all-meancov.txt and combined_host_prediction_to_genome_m90.csv.

Evaluating these temporally classified archetypes further, we next sought to link these to changes in host predictions, which revealed dominant host prediction abundances varying between archetypes (Fig 7). Archetype 2 consisted of viruses that predominantly targeted genera that made up ≤1% of the predictions in that archetype with only 4 different genera (*Winogradskyella, Pelagibacter, Joostella,* and *Flavobacterium*) having >1% of the host predictions. This is contrary to the other archetypes that possessed more host predictions that accounted for >1% of the viruses, perhaps because they were composed of so many more viral populations. Another notable observation was that archetype 1 held the largest proportion of phototroph targeting viruses, with viruses predicted to infect *Prochlorococcus_A* being the fifth most abundant host in the archetype (Fig 7). Archetype 1 mainly consisted of viruses peaking in abundance during the night with the largest proportion peaking at 20:00 hours. Our observations are further supported by bacterial data sampled during the same expedition that show *Prochlorococcus* bacteria having larger abundances during nighttime samples [54]. The correlated abundance of viruses with their hosts seen here with *Prochlorococcus* coincides with other studies that used transcripts and have similarly seen phototrophic targeting viruses being co-expressed with their associated bacteria [41,42,87]. The night peaking viral activity of SAR11 and *Prochlorococcus* have also previously been observed as we did at BATS [41]. Our archetype comparisons, show that viruses that target heterotrophs are the main ones showing diel patterns and that among our archetype 2 viruses that mainly peak in abundance during the day, the biggest targets were the genera *Winogradskyella, Pelagibacter, Joostella,* and *Flavobacterium* (Fig 7). As these genera are also found to be targeted in the other archetypes, it is possible that the difference here lies in a higher resolution of taxonomy for the bacteria being targeted.

## Discussion

In this study, we leveraged a high-resolution sub-daily temporal dataset from SUR and DCM waters to investigate spatial and temporal viral dynamics at BATS, a model “future” ocean given climate predictions. With respect to depth, we observed distinct differences in viral alpha and beta diversity across the depth-gradient, which were likely shaped by physicochemical features inherent to each depth such as light, temperature, oxygen, and chlorophyll concentration [61–63]. Additionally, we expanded upon our prior work to show how host predictions for depth-structured virus populations changed at BATS [52]. Temporally, however, the picture was different—viral community-scale metrics remained stable across the 112 hour time course, whereas differences could be observed at the viral population-level. Our observations that viral community structure remained stable across the time-series while viral population abundances varied, mirrors a broader pattern seen in monthly-sampled, longer timescale studies [34–36,38]. These results reinforce the idea that viral populations are dynamic even when community level metrics appear stable, with implications for host interactions, gene flow and downstream ecological impacts. Suggesting that virus-host interactions may maintain stability but with underlying evolutionary dynamism happening at a finer resolution through continuous changes among viral populations, driven by ongoing selection pressures from hosts and the environment. Exploration of these population-level differences revealed diel rhythmicity with machine learning-enabled methods [60] resolving such populations into one of three archetypes. Biologically, these archetypes differed in hosts predicted, viral taxonomy, and gene content.

By capturing viral abundances across depth and time, we uncover a complex and temporally structured viral community that mirrors host activity and environmental variation. The variation seen at the population-scale suggests that viral–host interactions are governed by more than abundance alone, likely shaped by biogeochemistry, host genome architecture, and ecological context [33]. Though not yet accomplished anywhere, future BATS efforts would benefit from many recent innovations that could layer in additional data, measurements, and interpretive frameworks. Long-read sequencing or single virus genomics could improve the capture of niche-defining genomic islands and microdiverse populations that are often missed by short-read assembled viromes [88,89]. Viral identification could be improved with the incorporation of additional methods such as GeNomad [58] once guidelines on how to effectively combine outputs on multiple tools without increasing the risk of increased contamination or decreased precision in true-positives are completed. Host predictions could be improved in a targeted manner through viral tag and grow experiments [90] or community-wide through DNA Hi-C proximity ligation sequencing, the latter assuming appropriate controls and analyses [91]. Measurements to assess activity, via metatranscriptomes, metaproteomes, metabolomes, and/or isotopic probing [92], could distinguish integrated past virus functioning emergent from viral particle sequencing from presumably real-time virus infection conditions. With better datasets, one could also expand the biological “players” in the story, for example, by exploring the interplay between viruses and other mobile genetic elements to better inform the biological entities interrupting and/or carrying key metabolic reactions [93]. Additionally, modeling frameworks that predict viral roles in community metabolisms [94] and experiments that focus on phenotyping the ecological costs of resistance [17] could provide and test specific hypotheses about viral roles in biogeochemistry that will lead to better incorporation of viruses into predictive models. Again, such a diverse toolkit has not yet been applied anywhere, but does aspire to more holistically capture true biological integration across molecular and organismal scales that dictate ecosystem functioning.

On the whole, viruses impact marine ecosystems via lysis, gene transfer, and metabolic reprogramming during infection [95]. The BATS time series in the Sargasso Sea, with its multidecadal records of biogeochemical and physical data, provides opportunity to assess how viruses contribute to and are shaped by ocean warming, stratification, and shifting nutrient regimes [96], particularly as its gyre expands, warms, and acidifies [49]. Our work focuses on establishing baseline viral genomic datasets across two depths at BATS, where these observations provide glimpses into host-driven structuring of virus communities. Such observations show enrichment of viruses targeting *Prochlorococcus_B* and SAR11 clade bacteria in DCM and SUR respectively, along with genes predicted to be associated with said taxa such as *psbA* for *Prochlorococcus_B*. Along with this, we also document short-term temporal changes that manifest not in community-aggregated measures, but instead at the population-level where we revealed the diversity and potential functioning of viruses that differentially occupy day- or night-peaking niches. These short-term temporal changes not only illustrate that heterotrophic targeting viruses make the majority of our diel signal, but that 94.09% of these diel viruses peak in abundance during the night. Placing such short-term change into the context of longer-term changes already known for marine bacteria [97] will be critical to better incorporate viral ecology and roles into long-term ocean observing systems that seek to better predict future ocean functioning.

## Methods

### Sample collection

Samples for this project were collected under Special Permit SP190906 from the Government of Bermuda Department of Environment and Natural Resources. Sample collection took place on the RV *Atlantic* Explorer (cruise AE1926) and coordinates of samples can be found within S1 Table. Sea water samples were collected once every 4 hours in the surface, SUR, or 12 hours in the DCM for 112 hours resulting in 39 samples (29 SUR, 10 DCM). A surface buoy with an underwater drogue, at 30 m depth, was deployed to allow us to follow the same parcel of water for the entire study. Water samples were collected using a CTD-rosette equipped with 24 × 12-l Niskin bottles. Depths for each cast were chosen based on in situ CTD oceanographic parameters. Sampling started at 16:00 local time (GMT-3) from SUR waters at ~5 m, always in the stratified SUR mixed layer, and 105–120 m in the DCM mesopelagic layer. For each cast, the CTD was deployed to at least 500 m to collect data on physical water columns, measuring the water temperature, pH, and salinity. Measurements were binned to the nearest 0.5 m of depth to standardize across casts, then a Nadaray-Watson kernel smoothing filter with bandwidth of 5 db was applied to each variable to remove noise and spikiness. For each sample, 10 L of seawater were filtered through a 0.2 µm Millipore filter (CAT number: GPWP14250;LOG number ROBA92539) to minimize cells, and iron chloride flocculation [53] was performed to concentrate the viruses in the 0.2 µm filtrate. The virus-concentrated iron hydroxide flocs were then filter-captured using a 1 µm polycarbonate filter and stored damp on the filter at 4 °C until resuspended in ascorbate-EDTA buffer and processed to DNA.

### Library preparation and sequencing

For each sample, half a filter, corresponding to ~5 L of seawater was resuspended in 7.5 mL of ascorbate-EDTA resuspension buffer to capture the concentrated viral particles. All samples were then treated with DNase diluted 1:40 in DNase reaction buffer. DNase was then inactivated by adding 0.1 M EDTA then 0.1 M EGTA. Viruses were concentrated by spinning each sample through a 15 mL 100 kDa Amicon filter and resuspended in ascorbate-EDTA buffer (0.1 M EDTA, 0.2 M Mg, 0.2 M ascorbic acid, pH 6.0) in total of less than 1 ml. DNA was then extracted using Wizard PCR Preps DNA Purification Resin and Mini-columns (Promega, Cat. #A7181 and A7211, respectively) [98]. Between 216 and 620 ng of DNA (355 ng on average between samples) was provided to the Joint Genome Institute for sequencing. Shotgun metagenomic sequencing at JGI was performed on an Illumina platform, resulting in an average of 144M reads per sample (~9.8 × 10^7^ to 2.6 × 10^8^ sequencing reads per sample).

### Quality check, trimming, and assembly

Data processing and metagenomic analysis were conducted on the Ohio Supercomputer Center [99]. Reads went through a quality check using BbDuk (https://sourceforge.net/projects/bbmap/). Low-quality reads, adaptors, and Phix174 reads were removed (ktrim = r minlength = 30 k = 23 mink = 11 hdist = 1 hist2 = 1) and reads were trimmed (qtrim = rl maq = 20 maxns = 0 minlength = 30 trimq = 20). Four samples were resequenced due to low-quality reads (T4D, T52S, T88S, T28D). Fastqc identified T28D as having a low read quality, so it was removed from the study. Resequenced samples had an average sequencing depth of about 381M reads per sample, due to this, resequenced samples had their cleaned reads subsampled using bbmap 38.96 (https://sourceforge.net/projects/bbmap/) to an even depth of 62,381,078 in the SUR and 68,082,965 in the DCM which is the median depth value for clean reads in the rest of the samples in these regions. Samples were assembled using MegaHit [100] with default options.

### Viral identification

Contigs from MegaHit were processed using Virsorter 2 SOP version 2.2.3 [55]. Briefly, all samples were first processed via Virsorter2 (–keep-original-seq –include-groups sdDNAphage, ssDNA –min-length 5,000 min-score 0.5). Then CheckV v0.8.1 [101] was used along with Virsorter2 outputs to curate viruses following the parameters in the Virsorter 2 SOP [55]. After curation, the pipeline identified 228,013 viruses which were then clustered with CheckV clustering v0.8.1 [101]. 48,428 viral populations greater than 5 kb and 14,634 greater than 10 kb were clustered at 95% average nucleotide identity and 80% coverage. A viral population is defined here as a group of viruses of the same species [47].

Additionally, all contigs from MegaHit were processed using GeNomad [58] end-to-end for comparison with VirSorter2 results.

### Read mapping

CoverM v0.6.1 was used to map quality-trimmed reads to viral populations (https://github.com/wwood/CoverM; –output-format dense, min-read-percent-identity 0.95, min-read-alignment-percent 0.75, min-covered-fraction 0.75, -m trimmed_mean). Reads were retained if they had ≥95% identity, ≥75% alignment, ≥75% coverage, and the trimmed mean was used to remove the top 5% and bottom 5% depths [70]. CoverM generates BAM files which are then summarized into coverage values that are then normalized by dividing them with read depth from the quality-trimmed reads per sample and multiplying by 1,000,000,000.

### Diversity calculations and statistical analyses

Diversity estimates were based on the normalized relative abundance tables generated via read recruitment. The alpha (Inverse Simpson) and beta (Bray-Curtis dissimilarity) diversity statistics were calculated using vegan on R (v 2.6.4) [102]. Read were log2 transformed before calculating Bray-Curtis (function vegdist, method = ‘bray’). Affinity propagation using APCluster on R was used to cluster samples into groups with similar viral relative abundance and community structure [103]. Communities were then divided into groups based on similarity for analysis and on depth and time of day for separate alpha/beta diversity analysis. Principal coordinate analysis (function cmdscale) and nonmetric multidimensional scaling (function metaMDS, permutations = 999) were ordination methods used on the Bray-Curtis dissimilarity matrices [104–106]. The statistical significance between viral communities was validated by comparing the within-community and between-community distances with MRPP and ANOSIM [107,108].

### Temporal analysis with the detection of rhythmic behavior in time series

For all datasets from BATS, diel periodicity was determined using the rank-based Jonckheere-Terpstra umbrella test implemented in the R RAIN package [85]. Normalized read abundance tables of the viral populations were observed for oscillating behavior across the 112-hour time series. Samples were first detrended (linear regression with respect to time was subtracted from time series) to increase power of rhythmicity detection using the detrend function in the R pracma package v.2.4.4 [85]. RAIN was run with the options method = ‘deltat = 4, period = 24’ for the surface samples to establish four-hour sampling periods and a 24-hours time period to observe for oscillation. Viral populations within the SUR samples had the options method = ‘measure.sequence = c(1,1,1,1,1,1,1,1,1,1,1,1,1,0,1,1,1,1,1,1,1,1,0,1,1,1,1,1,1)’ to account for sample “T52_S” and “T88_S,” that were excluded from analysis. Samples from the DCM were run with the options method = ‘deltat = 12, period = 24′ to establish 12-hour sampling periods and a 24-hour time period to observe for oscillation. Viral populations within the DCM samples had the options method = ‘measure.sequence = c(0,1,0,1,1,1,1,1,1,1)’ to account for samples “T4_D” and “T28_D” that were excluded from analysis. After RAIN implementation, the Benjamini–Hochberg false discovery rate control procedure was implemented to assess significance at the P = 0.05 level for each data type.

### Calculation of peak rank time

To estimate the mean peak time for viral populations, a rank-based heuristic was calculated. For a given viral population, the abundance at each time point was ranked. The ranks from all measurements were then averaged and the peak mean rank time was defined as the time with the highest average, where ties were summarized as the center between tied times [84].

### Clustering analysis

Detrended diel time series were scaled to make data dimensionless to reduce impact of magnitude on euclidean distance matrices. Euclidean distance matrices had a Hopkins statistic calculated to determine the meaningfulness of clustering where a value of *h = 0.71* was found, indicating data structure cannot be explained by random distribution of distances. To determine the best clustering method we employed hierarchical clustering (implemented by the hclust function in the R stats package v.4.1.1), medoid clustering [109], and training of SOMs [110]. These clustering methods were then evaluated for best fit using the Calinski-Harabasz metric and average silhouette distance. SOM was selected as the best fit clustering method on the basis of identifying the ‘elbow’ in decreasing average silhouette width to initially select three as the operational number of clusters. Fits were assessed in more detail for two and four clusters as well.

### Taxonomic classification

Prodigal v2.6.3 [111] was used to predict proteins from the viral populations greater than 10 kb. From the 15,197 viral populations greater than 10 kb, prodigal predicted 360,993 proteins. vConTACT 2 version 0.11.1 [112], was used to classify viral genomic sequence data. This specific tool is designed to cluster and provide a taxonomic context of metagenomic sequencing data. VConTACT2 results were then read into cytoscape v3.10.1 for visualization of the network following the protocol published on protocols.io [112].

### Functional annotation and auxiliary metabolic genes (AMGs)

AMGs were annotated using DRAMv v1.4.0 (min-contig-size = 10,000, --skip_trnascan; [113]). AMGs with a score of 1–3 and assigned to a metabolic module, and/or to a previously described AMG (flags M, K, or E) were selected as putative AMGs [70,114]. AMGs were then curated for a conservative catalog using previously established rules [70] where an AMG was only kept if located within virus regions called by checkV and/or found on a contig with a score ≥0.95 assigned by VirSorter2 [70] we screened for non-virus regions by checking for sequences adjacent to phage genome ends, including tRNA regions and inverted/direct repeats. tRNA regions were detected by tRNAscan-SE (v1.2.3; [115]) using general tRNA models. EMBOSS (v6.6.0; [116]) with standard qualifiers and -scircular1 for circular virus contigs was used to detect inverted and direct repeats in contigs. For AMGs with predicted phage ends we used the criteria mentioned in the aforementioned established rules for a conservative catalog [70] and removed AMGs found on viral contigs containing mobile genetic elements and other genes that may facilitate the random integration of microbial metabolic genes using the keywords transposons, lipopolysaccharide islands (glycosyltransferase, nucleotidyl transferase, carbohydrate kinases, and nucleotide sugar epimerase), endonucleases, integrases, or plasmid stability genes [70]. We then filtered for those containing pathway information from KEGG bringing our conservative catalog from 887 AMGs to 349 that contain a KEGG identifier. The conservative AMG catalog was mapped to metabolic pathways using Anvi’o v8 [117]. Metabolic pathways were visualized in KEGG Metabolic Pathways (map 01100) with iPath 3.0 [118] based on the presence of AMG-carrying viruses across depth layers and their diel signals. Pathways with default stepwise completeness less than 0.75 were considered incomplete.

### Identifying hosts using iPHoP with the standard database

IPHoP v1.3.2 [65] was used to predict hosts from viral populations >5 kb. Hosts were first identified using the standard database using the default commands.

### Identifying hosts using iPHoP with a custom database

A custom database was also created using 89 MAGs from a previous BATS study done in July of 2017 [52]. Following the protocol on iPHoP’s github page (https://bitbucket.org/srouxjgi/iphop/src/main/), GTDB [119] was used to assign taxonomy to the MAGs, which were then added to the standard iPHoP database to create a custom database. GTDB was run separately identifying bacteria with the outgroup *p_Patescibacteria* and archaea with the outgroup *p_Altiarchaeota*. Identified MAGs were then added to the standard iPHoP database using the add_to_db command. Identifying hosts from the custom database was done using the predict command.

### Sample collection from which MAGs were identified

The custom database uses MAGs identified from Warwick-Dugdale, 2017 dataset [52]. These MAGS were derived from 12 samples at the BATS station (31°40′N, 151 64°10′W) during dusk (~19:00 local time) and dawn (~06:00 local time), from the depths of 80 and 200 m, over a period of four consecutive days from the 8th to 11th of July 2017.

## Supporting information

S1 Fig VirSorter2 and Genomad comparison. Euler plot of viral identification between Genomad and VirSorter2 tools. Each tool was run on whole dataset, filtered to 5 kb sequences, then results were combined and clustered. Clustered sequences were then checked for if sequences that make up each cluster were exclusively from Genomad, VirSorter2, or shared.
(TIF)

S2 Fig Affinity propagation of depth clusters through viral relative abundance. Affinity propagation heatmap of Bray-Curtis dissimilarities compared between viral communities at each time point. Clustering generated through affinity propagation of the Bray-Curtis data was significantly different (Multi-Response Permutation Procedure [MRPP], *p* = 0.001). Clusters within affinity propagation heatmap are distinguished by a colored bar bordering the heatmap and dissimilarity is distinguished by the Bray-Curtis³ color scale. The data underlying this Figure can be found in the zenodo repository (see Data availability) within file all-meancov.txt.
(TIF)

S3 Fig Principal Coordinates Analysis (PCoA) of viral community between SUR and DCM samples. PCoA of viral community was performed on Bray-Curtis dissimilarities of viral populations in SUR and DCM samples. Each point represents a sample from SUR or DCM indicated by the color of the dot. Ellipses indicate the 95% confidence interval around the centroid of each region, calculated assuming a multivariate normal distribution. Communities were significantly different (Multi-Response Permutation Procedure [MRPP], *p* = 0.001, a PERMANOVA test *p* = 0.001). The data underlying this Figure can be found in the zenodo repository (see Data availability) within file all-meancov.txt.
(TIF)

S4 Fig Alpha diversity compared against Global Ocean Virome 2 (GOV2) dataset ecological zones. Difference of Inverse Simpson’s diversity indices SUR-DCM is plotted for our BATS samples (from October, 2019) along with data for the ecological zones from the GOV2 data [47] of TT (Temperate and tropical epipelagic waters), ANT (Antarctic water), and ARC (Arctic waters). The data underlying this Figure can be found in the zenodo repository (see Data availability) within file Normalized_Viral_Abundances_ALL_95ANI_5kb_ECOLOGY.txt and all-meancov.txt.
(TIF)

S5 Fig Viral Families of SUR diel and non-diel viral populations. Alluvial plot of viral taxonomy for viral populations present at the family level. Height, order, and pie chart are as described in Fig 3. The data underlying this Figure can be found in S2 Data and in the zenodo repository (see Data availability) within file all-meancov.txt.
(TIF)

S6 Fig Principal Coordinates Analysis (PCoA) of beta-dispersion among three viral archetypes. Beta-dispersion PCoA was performed on euclidean distance of surface viral populations based on abundance data across time series using S12 Table, reordered by archetype information in S13 Table. Each point represents a viral population colored by archetype, with length of line signifying the distance to median of multivariate dispersion. Archetypes are significantly different from one another using PERMANOVA and Tukey’s test (*p* < 0.001). The data underlying this Figure can be found in the zenodo repository (see Data availability) within file all-meancov.txt.
(TIF)

S1 Table Metadata for BATS 2019 Cruise. Table of metadata collected for dataset with regards to latitude, longitude, salinity, temperature, chlorophyll, and depth.
(XLSX)

S2 Table Raw and clean read numbers for samples. Table of number of raw reads and bases along with clean reads and bases for each sample. Sample T28_D is crossed out due to low sequencing depth from the sequencing run.
(XLSX)

S3 Table Sample data across viral identification pipeline. Table of number of reads in files throughout the VirSorter2 viral identification pipeline. Starting at sample read depth and ending with checkV clustering and filtering to 10 kb size. Outlier samples are highlighted in different colors.
(XLSX)

S4 Table BATS MAGs GTDB-tk summary file. Table containing the MAGs used to form the custom iPHoP library for host predictions.
(XLSX)

S5 Table Conservative filtered AMGs annotations. Table of annotations for all AMGs that passed the conservative filtration process and possess a KEGG identifier.
(XLSX)

S6 Table Diel AMG annotations. Table of annotations for all conservative AMGs found within viral populations classified as diel.
(XLSX)

S7 Table Non-Diel AMG annotations. Table of annotations for all conservative AMGs found within viral populations classified as non-diel.
(XLSX)

S8 Table Non-Diel AMG modules. Table containing the module and pathway information for non-diel AMGs based on Anvi’o classification using anvi-estimate-metabolism that takes the ko_id from an AMG and the gene identifier to determine the pathway and module.
(XLSX)

S9 Table Diel AMG modules. Table containing the module and pathway information for diel AMGs based on Anvi’o classification using anvi-estimate-metabolism that takes the ko_id from an AMG and the gene identifier to determine the pathway and module.
(XLSX)

S10 Table Host predictions with associated AMGs for depth groups. Table containing conservative AMGs organized by host prediction for the viral population it was found in along with the number of instances that AMG was found in that predicted host for each depth. The table also contains the ko_name, symbol, and pathway information according to KEGG.
(XLSX)

S11 Table Host predictions with associated AMGs for diel groups. Table containing conservative AMGs organized by host prediction for the viral population it was found in along with the number of instances that AMG was found in that predicted host for either diel or non-diel viral populations. The table also contains the ko_name, symbol, and pathway information according to KEGG.
(XLSX)

S12 Table Diel viral populations. Table containing the viral populations considered as diel and their trimmed_mean counts from CoverM across the dataset.
(XLSX)

S13 Table Archetype information. Table containing viral populations that were diel along with the archetype (cluster) they belong to and the time block they peaked on average throughout the study (time_rank). The x and y columns pertain to coordinates for plotting.
(XLSX)

S1 Data Table of depth profile of chlorophyll and temperature down to 1,000 m depth for 2019 BATS cruise. (XLSX)

S2 Data Table of viral taxonomy from vConTACT3 using viral populations that are 10 kb or larger. (XLSX)

## Abbreviations

AMGs: auxiliary metabolic genes
BATS: Bermuda Atlantic Time Series
DCM: deep-chlorophyll maximum
MAGs: metagenome-assembled genomes
SOM: self-organizing map
SPOT: San Pedro Ocean Time
SUR: surface
vOTUs: virus operational taxonomic units
